# Non-Woven Fabric Thermal-Conductive Triboelectric Nanogenerator via Compositing Zirconium Boride

**DOI:** 10.3390/polym16060778

**Published:** 2024-03-12

**Authors:** Xin Wang, Jinming Liu, Haiming Chen, Shihao Zhou, Dongsheng Mao

**Affiliations:** 1 School of Materials Science and Chemical Engineering, Ningbo University, Ningbo 315211, China; wangxin12@nimte.ac.cn; 2 Key Laboratory of Marine Materials and Related Technologies, Zhejiang Key Laboratory of Marine Materials and Protective Technologies, Ningbo Institute of Materials Technology and Engineering, Chinese Academy of Sciences, Ningbo 315201, China; liujinming@nimte.ac.cn (J.L.); zhoushihao@nimte.ac.cn (S.Z.); 3 Department of Materials Science and Engineering, Zhejiang University of Technology, Hangzhou 310014, China

**Keywords:** triboelectric nanogenerator, thermal conductivity, polyurethane, zirconium diboride, composites

## Abstract

With the vigorous development of the Internet of Things, 5G technology, and artificial intelligence, flexible wearable sensors have received great attention. As a simple and low-cost power supply in wearable sensors, the triboelectric nanogenerator (TENG) has a wide range of applications in the field of flexible electronics. However, most polymers are thermally poor conductors (less than 0.1 W/(m·K)), resulting in insufficient heat dissipation performance and limiting the development of TENG. In this study, a high-performance non-woven fabric TENG with strong thermal conductivity (0.26 W/m·K) was achieved by introducing ZrB_2_ into the polyurethane (PU) matrix. The excellent output performance with an open circuit voltage (V_oc_) of 347.6 V, a short circuit current (I_sc_) of 3.61 μA, and an accumulated charge of 142.4 nC endows it with good sensitivity. The electrospun PU/ZrB_2_ composites exhibit excellent sensing performance to detect body movements in situ, such as pressing, clapping, running, and walking. Moreover, the generated power can light up 224 LED bulbs as a demonstration of self-powering ability.

## 1. Introduction

With the vigorous development of the Internet of Things, researchers have developed intelligent flexible electronic devices that integrate multiple functions, which play an important role in health assessment, human–computer interaction, and biomedicine [1,2,3,4]. Therefore, stretchable, compressible, and twistable flexible electronic devices, which have advantages over traditional sensors, have aroused widespread attention [5]. However, the existing flexible electronic devices often require external power to maintain their operation, which undoubtedly increases the complexity and production difficulty of the sensing system as well as sacrificing the flexibility [6]. The triboelectric nanogenerator (TENG), which has the advantages of low cost, high efficiency, and sustainable mechanical–electrical energy conversion, is a good candidate that can be regarded as a flexible electronic device [7,8].

Electrification as one of the contact electrification phenomena often occurs when combing hair, wearing clothes, and opening doors [9]. The charges aroused by friction can thus be collected by electrodes and stored, which is the working principle of TENG [10]. Based on the coupling of contact electrification and electrostatic induction, TENG can collect and convert various low-frequency mechanical energies into electric ones. Additionally, the TENG can also be used as a self-driven sensor, that is, the voltage and current output are used to describe the mechanical triggering, e.g., in human–computer interactions, electronic skin, disease detection, and so on [11]. 

Currently, introducing inorganic fillers with a high dielectric constant is the mainstream strategy to improve the output performance of TENG, which can provide enhanced charge storage for the device [12]. Chen et al. modified the friction material (polydimethylsiloxane, PDMS) of TENG by filling high dielectric constant nanoparticles. The film is composed of 10 wt% SrTiO_3_ nanoparticles and 15 vol% voids. With the combination of the enhancement of permittivity and production of pores in the PDMS film, the charge density of ~19 nC cm^−2^, open-circuit voltage of 338 V, and power density of 6.47 W m^−2^ are obtained at a working frequency of 2.5 Hz, with the optimized film consisting of 10% SrTiO_3_ nanoparticles (~100 nm in size) and 15% pores in volume, which gives over five-fold power enhancement compared with the nanogenerator based on the pure PDMS film [13]. Wang et al. added polyethylene glycol (PEG) additives and polytetrafluoroethylene nanoparticles (PTFE-NPs) into thermoplastic polyurethane (TPU) as triboelectric layers. Through optimizing the dielectric constant of the composite, the injected charge density and internal capacitance of the TENG are significantly enhanced, thus synergistically boosting the output power and reducing the impedance of the TENG. The optimal output power reaches 16.8 mW at an external resistance of 200 kΩ, showing a 17.3 times enhancement in output power and a 90% decline in matching impedance [14]. 

The triboelectrification effect is also related to the surface morphology and structure of the material. Therefore, by designing the morphology and structure of the material, the purpose of increasing the friction contact area to increase the surface charge density can be achieved [15]. Kim et al. fabricated ordered microstructures on the surface of PDMS by ultrafast laser irradiation [16]. Compared with untreated PDMS, the output power of PDMS fabricated with 29 MW laser power increased by more than twice. Cheng et al. used argon plasma to etch the PDMS surface to introduce a microstructure [17]. When the plasma power was 90 W and the treatment time was 5 min, many uniformly distributed micropillars appeared on the PDMS surface. The output performance of TENG after treatment was 2.6 times that of untreated TENG. Zhu et al. used nanoparticles to modify the surface of the material, and the concave–convex surface formed by nanoparticles provided a larger contact area than the gold film, thereby improving the output performance of TENG. The record high power output for the triboelectric nanogenerator is attributed to optimized structure, proper materials selection, and nanoscale surface modification [18]. Choi et al. reported a facile and cost-effective route for fabricating highly efficient triboelectric energy harvesters via formation of an artificially well-tailored interlocked interface with a nanostructured Ni electrode and PDMS. The interlocked interface formed between the nano-pillar Ni electrode and nano-pillar PDMS composite thin film effectively enhanced the triboelectricity of a TENG by increasing the surface contact area and contact time, related to the frictional forces [19]. 

The output performance of TENG is also closely related to the chemical properties of the two kinds of electric materials. The output performance of TENG can be improved by changing the properties of the materials through chemical modification. Functional group grafting is a simple and effective method to prepare tunable high-performance TENG by simply introducing electron-withdrawing groups or electron-donating groups on the surface of the electrification material. Shin et al. modified the triboelectric properties of polymer surfaces by chemical functionalization at the atomic level. The surface of polyethylene terephthalate (PET) was functionalized with halogen-containing benzene-derivative molecules or aminated molecules. By using this method, an adjustable triboelectric output power density was achieved in TENG [20]. Functional group grafting is a simple, economical, and easy method, which can change the surface chemical properties of friction materials by breaking and forming new chemical bonds on the surface of friction materials. However, since functional group grafting is only carried out on the surface of the friction material, rather than deep inside the friction material, the grafting effect may fail if the surface of the friction material is worn. 

Adding ions directly on the surface or inside of the friction material by ion implantation can enhance the surface charge density, thereby improving the output performance of TENG. Compared with functional group grafting, ion implantation can modify the material internally [21]. Fan et al. reported a modification method based on ion implantation technology to generate these specific chemical bonds to achieve ultra-high negative triboelectric polymers, which greatly improves the triboelectric properties of materials. The modified fluorinated polymer exhibits ultra-high negative friction electrode properties, and its surface charge density is increased by 4~8 times compared with the original sample. At the same time, the stronger polarity doubles the dielectric constant and further improves the energy storage density of the material [22]. Ion irradiation is an irradiation method widely used in metals, superconductors, semiconductors, and other materials. Its scope of application is controllable, the ion dose is adjustable, and the treatment result is uniform [23]. Li et al. used low-energy ion irradiation to adjust the chemical structure and functional groups of polymers at the molecular level to achieve surface modification of materials. The polyimide (Kapton) film modified by ion irradiation exhibits some excellent properties, such as high surface charge density, excellent stability, and ultra-high electron supply capacity [24].

However, Joule heating is inevitably generated simultaneously in electrification. Polymer composites are widely used in various occasions due to their advantages of good flexibility, low density, good insulation, low cost, corrosion resistance and easy processing. Triboelectric nanogenerators are mainly composed of polymer composites. However, for most polymers, phonon heat conduction is the main heat conduction pathway. Because the amorphous structure and vibration of macromolecular chains in polymers can cause a large amount of phonon scattering, most of the neat polymers are thermal insulators or relatively poor thermal conductors. The thermal conductivity of polymers is usually less than 0.1 W/(m·K), resulting in insufficient heat dissipation and a short lifespan of TENG [25]. Especially in the wearable field, the temperature rise caused by heat not only makes it uncomfortable but also affects the output performance. Therefore, it is necessary to pay attention to the heat dissipation capacity of TENG while improving the output performance of TENG [26]. Recently, researchers have introduced auxiliary thermoelectric generators (TEGs) to remove the Joule heating [27]. However, the introduction of TEGs increases the complexity of the structure and the cost, but lacks the wear resistance of the whole generator equipment. 

According to research, two methods are mainly used to improve the thermal conductivity of polymers: one is intrinsic modification based on the molecular structure to regulate the thermal conductivity of polymers, that is, a series of regulations of the polymer molecular chain structure, forming a regular and orderly structure inside the polymer matrix to improve its crystallinity or orientation, and then improve the thermal conductivity of polymer materials. The high thermal conductivity polymer obtained by this method is called intrinsic thermal conductivity material [28]. Zhu et al. found an increase in λ value from W/(m·K) (unstretched) to 51 W/(m·K) by heat-stretching Spectra S-900 ultrahigh-molecular-weight polyethylene (UHMWPE) microfiber. It was found, using X-ray diffraction, that the crystallinity of UHMWPE decreased from 92% to 83% after stretching, while the crystallite size and crystallite orientation did not change. The result from polarization Raman spectroscopy showed that the amorphous structure became more aligned after stretching, suggesting that the significant increase in the thermal conductivity of the polymer was attributable to the enhanced alignment of the amorphous chains. When the polymer has a higher crystallinity, it will generally possess a better thermal conductivity [29]. Mehra et al. reported the introduction of short-chain poly (ethylene glycol) (PEG) into long-chain poly (vinyl alcohol) (PVA) to improve the λ value of the polymer by forming new thermal conduction pathways through hydrogen bonding interactions between the two polymers. The number of thermal bridges increases with the continuous addition of PEG molecules, and at optimum loading (1:9 for PVA:PEG), a well-established thermal conductivity path is formed in the polymer, and the λ value of the PEG–PVA sample is enhanced to 1.6 times that of pure PVA films. The addition of PEG breaks the random intermolecular attraction of the PVA polymer chain, thus establishing ordered and homogeneously distributed hydrogen bonds and reducing phonon scattering, promoting thermal conductivity within the polymer [30]. However, this method is relatively complex, time-consuming, costly, and not commonly used. 

In contrast, the other method is relatively simple, with low cost, has strong controllability, and the heat-conduction effect is significantly improved. Therefore, the introduction of nanofillers with excellent thermal conductivity into the polymer matrix is a common method to improve the heat transfer performance of the polymer. The nanofillers include carbon materials, ceramic fillers, and metal fillers [31]. Among them, two-dimensional boron nitride nanosheets (BNNs) are an attractive choice for preparing insulation and thermal conductive composites due to their ultra-high thermal conductivity and excellent electrical insulation properties [32,33]. Cao et al. prepared polyimide (PI)/BNN composites by hot-pressing PI microspheres coated with BNNs. The microspheres facilitate the alignment of BNNs during hot pressing, avoiding serious agglomeration between BNNs. When the BNN content is 12.4 vol%, the thermal conductivity of the composite reaches 4.25 W/(m·K) [34]. Zhou et al. designed polyvinyl alcohol/polydopamine-modified boron nitride nanosheet (PVA/BNNS@PDA) nanocomposites with hierarchical structures by combining electrospinning, vacuum filtration deposition, and hot pressing. The modified BNNS@PDA improves the interaction between the filler and the polymer matrix while reducing the interfacial thermal resistance, resulting in superior thermal conductivity, excellent insulation, and perfect flexibility. The PVA/BNNS@PDA nanocomposites possess an ultrahigh in-plane thermal conductivity of 16.6 W/(m·K) at 35.54 wt% BNNS@PDA content. Even after 2000 folds, the nanocomposites do not experience any cracking, showing their ultrahigh thermal conductivity behavior [35]. Yu et al. reported a green, low cost, and high-efficient method to improve the thermal conductivity (λ) of carboxylated acrylonitrile-butadiene rubber (XNBR) composites via noncovalent modification of boron nitride (BN) via tannic acid (TA) chemistry. The noncovalent TA decorating on the surface of BN without deteriorating the surface structure of BN platelets ensures the high intrinsic λ of BN. Additionally, TA enhances the interfacial compatibility between the filler and matrix, as well as the formation of a thermally conductive path in the composites. The maximum through-plane λ is obtained by 30 vol% BN-TA-XNBR composite as 0.42 W/(m·K), which is 260% of that for pure XNBR (0.16 W/(m·K)) [36]. Although these methods effectively enhance the thermal conductivity of the polymer, the high-performance thermal conductive TENG has rarely been reported. 

Zirconium boride (ZrB_2_) is a good candidate for simultaneously fabricating the high-performance thermal conductive TENG due to its high dielectric constant (32.3) and thermal conductivity (23 W/m·K) [37,38,39]. The introduction of ZrB_2_ into the polymer can not only play an important role in the adjustment of the dielectric constant but also improve the thermal conductivity of the polymer to enhance its heat-dissipation capacity. Therefore, a high-performance TENG with strong thermal conductivity (0.26 W/m·K) was achieved by introducing ZrB_2_ into the polyurethane (PU) matrix. It has excellent output performance with an open circuit voltage (*V_oc_*) of 347.6V, a short circuit current (*I_sc_*) of 3.61 μA, and an accumulated charge of 142.4 nC. Finally, the PU/ZrB_2_ composites were electrospun into non-woven fabrics, which exhibit excellent sensing performance to detect body movements in situ, such as pressing, clapping, running, and walking.

## 2. Experiment

### 2.1. Materials

Zirconium boride (purity = 99%, diameter = 0.2–0.5 μm, density = 6.1 g/cm^3^) was purchased from Shanghai Aladdin Biochemical Technology Co., Ltd. (Shanghai, China). The methylene-bis(4-cyclohexylisocyanate) (HMDI, purity = 90%, Mn = 262 g/mol) was purchased from Meryer (Shanghai, China) Biochemical Technology Co. Ltd. The polycaprolactone diol (PCL, Mn = 530 g/mol) was purchased from Shanghai Macklin Biochemical Technology Co., Ltd. (Shanghai, China). The polyether amine (D400, Mn = 400 g/mol), butyl dilaurate (DBTDL), tetrahydrofuran (THF), N,N-dimethylformamide (DMF) were purchased from Shanghai Aladdin Biochemical Technology Co., Ltd. (Shanghai, China). All of these materials were used directly without further purification. 

### 2.2. Specimen Preparation

#### 2.2.1. Polyurethane Synthesis

PCL and D400 with preset contents were loaded into a three-neck flask and degassed at 60 °C for 30 min. Then, anhydrous DMF, DBTDL, and HMDI were added dropwise to react under nitrogen atmosphere for 24 h. Finally, the solution was poured into a Teflon dish before drying in a blast oven at 60 °C and in a vacuum oven at 60 °C, respectively. 

#### 2.2.2. Fabrication of Zirconium Boride/Polyurethane Composite

The synthesized polyurethane (PU) and zirconium boride were composited by a solution method. The PU was dissolved in DMF, and the zirconium boride was added to the PU solution via magnetic stirring. Ultrasonic treatment was then carried out to ensure its uniform dispersion. Finally, the solution was poured into a Teflon dish before drying in a blast oven at 60 °C and in a vacuum oven at 60 °C, respectively. The concentration of ZrB_2_ was set to be 0.9 vol%, 1.8 vol%, 3.6 vol%, 5.4 vol%, and 7.2 vol%.

#### 2.2.3. Preparation of Nanofiber Membrane

A mixed solvent (DMF:THF = 3:2) was used to dissolve the synthesized PU, and the zirconium boride was added to the PU solution via magnetic stirring; ultrasonic treatment was then carried out to ensure its uniform dispersion. Then, PU/zirconium boride nanofiber membrane was prepared by electrospinning. The voltage was 18 kV, the tip-to-collector distance was 16 cm, and the mass fraction of PU was 10%. The nanofiber membrane was finally dried at 60 °C to promote the removal of residual organic solvents. 

### 2.3. Characterization

The microstructure of the composites was characterized by scanning electron microscopy (SEM). The specimen fractured brittlely in liquid nitrogen and was coated with gold on the fracture surface. The dielectric properties were characterized using a broadband dielectric spectrometer (Concept80, NOVOCONTROL Technologies GmbH& Co.KG, Montabaur, Germany). A linear motor (LinMot, NTI AG, Spreitenbach, Switzerland) was used to simulate the mechanical motion, and a programmable electrometer (Keithley 6514, Tektronix Inc., Shanghai, China) was used to record the output performance of voltage, current, and charge by a computer based on the LabVIEW program. The thermal stability was characterized using a Thermal Gravimetric Analyzer (TGA, TG209F1, NETZSCH, Selb, Germany). The temperature rise rate was 10 °C min^−1^ in a N_2_ atmosphere. Nanocomposites were measured using a laser thermal conductivity meter (LFA457, NETZSCH, Selb, Germany).

## 3. Results and Discussion

### 3.1. Preparation and Morphology

**Figure 1:**
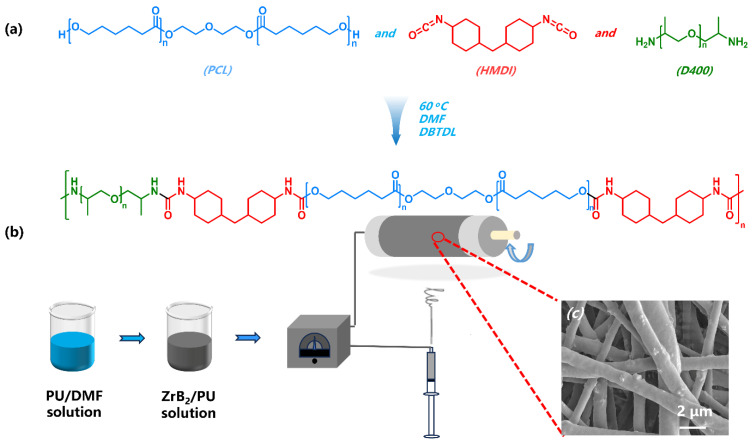
(**a**) Synthesis route of the polyurethane (PU) matrix. (**b**) Scheme of the non-woven fabric. (**c**) SEM image of non-woven fabric with 7.2 vol% ZrB_2_.

PU is considered an effective triboelectric layer mainly due to its extensive hydrogen bonding (H-bond) [40]. Therefore, implanting a large number of H-bond units will effectively improve the sensitivity of flexible sensors. Tailoring the configuration, type, and content of chain extenders from the perspective of molecular structure can significantly improve H-bond density. However, the introduction of chain extenders often requires at least a two-step method for synthesis, which increases the terms of cost and energy consumption. Here, we synthesized robust PU with high H-bond density through a one-step method by shortening the length of the soft segments; the synthesis route is shown in Figure 1a. Its strength can be as high as 60.93 MPa, as shown in Figure S1 in the supporting information. The mechanism behind the robust behavior has been studied in another work and also discussed in the Supporting Information. The synthesized PU was then compounded with pristine zirconium boride (ZrB_2_), and finally a nanofiber film was obtained through electrospinning technology. The preparation process of the nanofiber film is shown in Figure 1b. The morphology of the nanofibers is shown in Figure 1c. The fiber surface was smooth and the average diameter was 0.9 μm.

**Figure 2:**
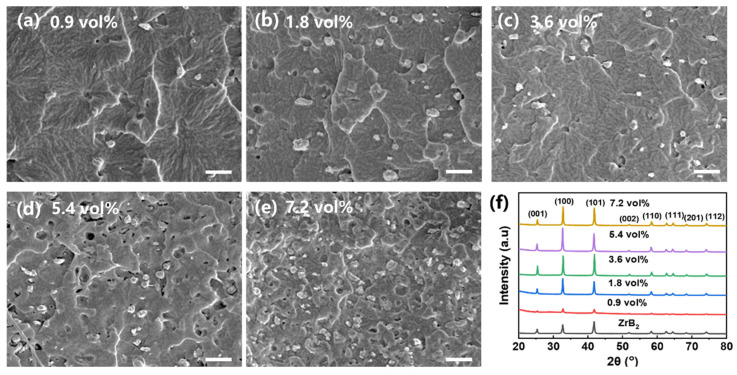
SEM images of PU/ZrB_2_ composites with different nanofiller contents; the scale bar is 5 μm: (**a**) 0.9 vol%; (**b**) 1.8 vol%; (**c**) 3.6 vol%; (**d**) 5.4 vol%; (**e**) 7.2 vol%. (**f**) XRD profiles of PU/ZrB_2_ composites.

Commonly, the dispersion of nanofiller in polymeric matrix is the first issue that needs to be addressed, because the significant aggregation of nanofiller caused by the nano effect would deteriorate macro-performance. Thus, the size of ZrB_2_ used in this work was set to be 200–400 nm to avoid massive aggregation. The SEM images in Figure 2 demonstrate that the ZrB_2_ particles are dispersed uniformly in the PU matrix regardless of the filler content, implying the solution method used in this work can significantly promote the dispersion of ZrB_2_ in the PU matrix. Furthermore, the solution method keeps the crystalline structure of ZrB_2_ from being destroyed; as shown in Figure 2f, all PU/ZrB_2_ composites show the same diffraction peaks compared with the pristine ZrB_2_ particle.

### 3.2. Thermal Conductivity

**Figure 3:**
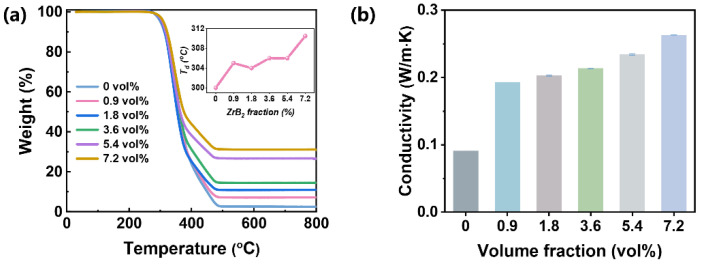
(**a**) TGA curves and the decomposition temperature (*T_d_*) of the PU/ZrB_2_ composites. (**b**) Thermal conductivity of the PU/ZrB_2_ composites.

Thermal stability and conductivity of the triboelectric layers are important properties for high-performance TENGs because the charger accumulation on triboelectric surface layers results in a local high-temperature area. The thermogravimetric curves of such composites are shown in Figure 3a. The initial decomposition temperature (*T_d_*, at the weight of 95%) increases from 300 °C to 310 °C with ZrB_2_, suggesting significantly improved thermal stability by adding ZrB_2_. The thermal conductivity of these composites is shown in Figure 3b; it increases from 0.091 W·m^−1^·K^−1^ (0 vol%) to 0.263 W·m^−1^·K^−1^ (7.2 vol%) with an increment of 188%. For polymers, the thermal conductivity is determined by the coupling strength of adjacent clusters and the vibrational motion of atoms [41]. The molecular chains of polymer materials are difficult to move freely at low temperatures. In addition, the disordered entanglement of molecular chains and the uneven distribution of molecular weight aggravate phonon scattering, resulting in low thermal conductivity of the polymer. For ZrB_2_ composites, excessive ZrB_2_ nanofillers form a threshold permeability network in the PU matrix. Combined with the high thermal conductivity properties of ZrB_2_ fillers, it gives the composite excellent thermal conductivity. 

### 3.3. Breathable Triboelectric Nanogenerator

**Figure 4:**
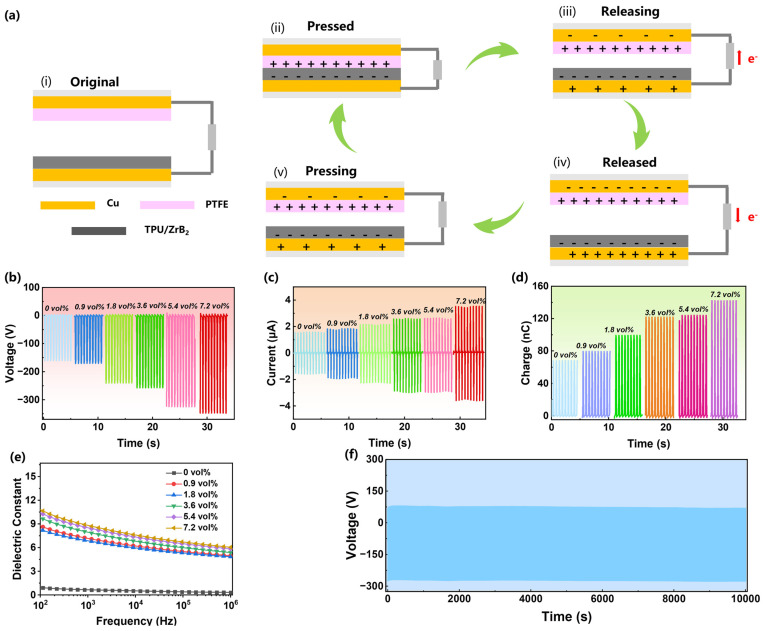
(**a**) Scheme of the TENGs working principle. (**b**) Voltage, (**c**) current, and (**d**) charges of TENGs. (**e**) Frequency dependence of the dielectric constant of ZrB_2_ composites. (**f**) Durability of the TENG by contact-separating the composite with ZrB_2_ content of 7.2 vol% and PTFE with a frequency of 2 Hz.

The triboelectric properties of these composites were characterized prior to electrospinning. Given the large number of hydrogen bonds in the PU matrix, it often becomes positively charged after contact with other substances. Therefore, typical polytetrafluoroethylene (PTFE) with electronegativity was chosen to contact the PU/ZrB_2_ composites, and copper foil was used as an electrode to collect the charge. Linear motors provided stable mechanical energy. Figure 4a shows the working principle of the vertical contact-separation TENG. When PTFE encounters the PU/ZrB_2_ composite, charges are induced at the interface due to the difference in electronegativity between them, namely the contact electrification (image ii in Figure 4a). However, the electrostatic charge generated on the surface cannot be derived immediately when the two are separated, since both PTFE and PU/ZrB_2_ are insulate materials, so the chargers will induce opposite chargers on the electrode surface to achieve balance (iii). At this time, connecting the two copper electrodes can generate a current in the circuit, and the induced charge increases as the separation distance between PTFE and PU/ZrB_2_ increases (iv). When PTFE and PU/ZrB_2_ approach, the decreases in induced charge on the electrode results in decreasing of the current and voltage at the same time (v) until the two electrodes reach equilibrium after they contact again, at which time the voltage and current decrease to zero.

Here, the effect of ZrB_2_ contents on the output performance of TENG are examined, as shown in Figure 4b–d. The open circuit voltage (*V_oc_*) increases from 160.6 V (0 vol%) to 347.6 V (7.2 vol%), the short circuit current (*I_sc_*) increases from 1.55 μA (0 vol%) to 3.61 μA (7.2 vol%), and the accumulated charge increases from 68.20 nC (0 vol%) to 142.4 nC (7.2 vol%), respectively, demonstrating an excellent promotion effect of ZrB_2_ on the output performance of TENG. It is believed that the excellent promotion is related to the dielectric properties of the composites. Figure 4e shows the broadband dielectric spectra of the composites. The dielectric constant increases from 0.9 (0 vol%) to 10.5 (7.2 vol%) at 100 Hz due to the high dielectric constant of ZrB_2_. A high dielectric constant has been proven to promote the accumulation of surface charges, thereby enhancing triboelectric charging capabilities [42]. Furthermore, taking the specimen of 7.2 vol% as an example, after 10,000 contact–separation cycles, it still maintains excellent and stable voltage output, indicating that the composite has excellent stability (Figure 4f).

**Figure 5:**
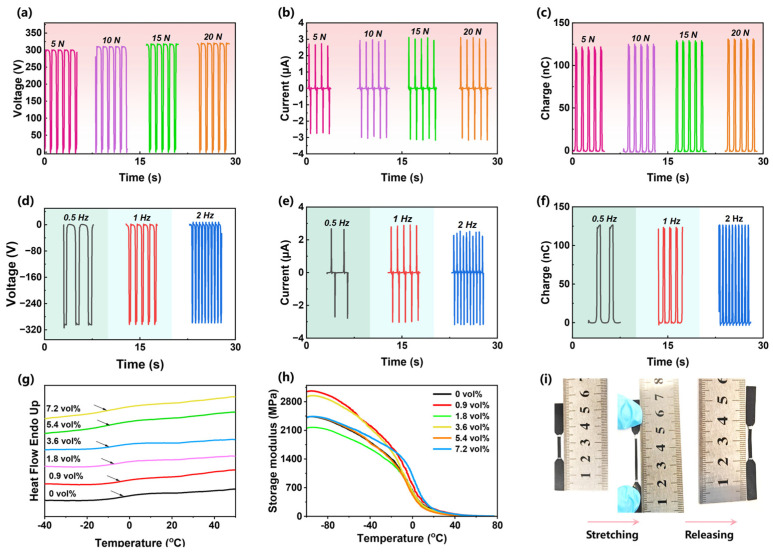
Stability of TENG fabricated by ZrB_2_. (**a**–**c**) Voltage (**a**), current (**b**), and charges (**c**) of the TENG performed at various forces. (**d**–**f**) Voltage (**d**), current (**e**), and charges (**f**) of the TENG performed at various frequencies. (**g**) DSC heating scans and (**h**) Storage modulus of PU/ZrB_2_ composites. (**i**) The recovery of 7.2 vol% ZrB_2_/PU composites once unloading.

Additionally, the TENG assembled from ZrB_2_/PU exhibits stability against applied force and frequency. Figure 5a–f shows the effects of different forces and frequencies on the *V_oc_*, *I_sc_*, and charge. They remain at 309.7~321.1 V, 2.63~3.03 μA, and 121.1~131.2 nC, respectively, regardless the force and frequency. Generally, both the force and frequency will promote the output performance of the TENG due to the enlargement of contact area. Here, the insensitivity of the TENG to force and frequency is considered to be mainly due to the excellent resilience of the composites. It can rebound well under different forces and frequencies, so the contact area does not change much when the force is applied. The DSC heating curves in Figure 5g prove that the glass transition temperature of the composites is between −10 and ~0 °C, that is, it is in an elastic state when performed at room temperature. The storage modulus in Figure 5h is 175.6 MPa at 20 °C, also showing the good elastic characteristics. Furthermore, the stretched specimen can fully recovery once releasing stress, demonstrating good elasticity (Figure 5i). 

**Figure 6:**
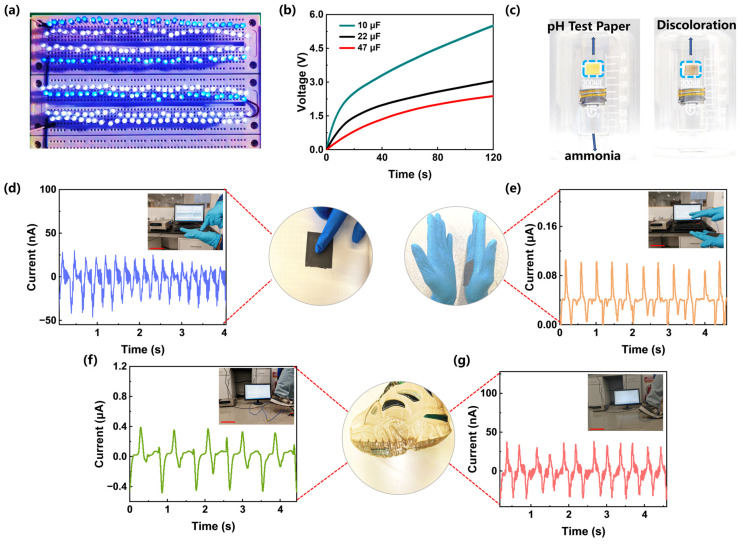
(**a**) Powering LED bulbs by the TENG (PU/ZrB_2_ (7.2 vol%)). (**b**) Capacitance charging curves for different capacities. (**c**) Permeability demonstration of PU/ZrB_2_ (7.2 vol%). (**d**–**g**) Body measurement monitoring of (**d**) pressing when it is attached to the arm, (**e**) clapping when it is attached to the palm, (**f**) running, and (**g**) walking when it is attached to the soles of feet (the scale bar is 25 cm).

The excellent mechanical-to-electrical energy conversion function can be further demonstrated by the non-woven fabrics achieved by electrospinning technology, which can be used to develop breathable wearable sensing devices. The generated power can light up 224 LED bulbs (Figure 6a). Moreover, the non-woven fabric still has excellent mechanical–electrical energy conversion ability. It can charge the capacitor of 10 μF, 22 μF, and 47 μF to reach 5.5 V, 3.0 V, and 2.3 V after contact-separating for 120 s. Additionally, although the strength of the spun filaments is lower than that of the bulk one, the strength of the non-woven fabric still reaches 16 MPa, which exceeds that of general polymer non-woven fabrics. It can be rubbed and stretched randomly without damage, showing excellent practical service capabilities (Figure S2, in the supporting information). The non-woven fabrics can last more than 60,000 cycles without any deterioration in voltage output, indicating that the composite has excellent stability (Figure S3, in the supporting information). Furthermore, the similar morphology between the one that before and after being contacted shows that the fabric structure has not been destroyed, which is mainly due to the good elasticity of the PU matrix (Figure S4, in the supporting information). Moreover, Figure 6c demonstrates its good gas permeability. Here, a beaker filled with ammonia was covered by the electrospun non-woven fabric. After 30 s, the pH test paper above it turned brown, indicating that the ammonia gas had volatilized and penetrated the non-woven fabric, proving that the non-woven fabric has good breathability.

Furthermore, Figure 6d–g shows the current variation when used to monitor body movements including pressing, clapping, running, and walking. No matter what form of movement, obvious current changes were produced. Taking clapping as an example (Figure 6e), when the palms are in contact, a forward current is induced, and a reverse one is generated when the palms are separated. Therefore, the wearable sensor prepared in this study has excellent motion-detection capabilities.

## 4. Conclusions

In this work, a high-performance non-woven fabric TENG with strong thermal conductivity (0.26 W/m·K) was achieved by introducing ZrB_2_ into the PU matrix. The excellent output performance with open circuit voltage (*V_oc_*) of 347.6 V, the short circuit current (*I_sc_*) of 3.61 μA, and the accumulated charge of 142.4 nC endow it with good sensitivity. The electrospun PU/ZrB_2_ composites exhibit excellent sensing performance to detect body movements in situ, such as pressing, clapping, running, and walking. Furthermore, the generated power can light up 224 LED bulbs as a demonstration to achieve the self-powering ability.

## Data Availability

Data are contained within the article.

## Supplementary Materials

The following supporting information can be downloaded at: https://www.mdpi.com/article/10.3390/polym16060778/s1.
