# Supplementary material for: Non-Woven Fabric Thermal-Conductive Triboelectric Nanogenerator via Compositing Zirconium Boride
Source: Polymers (Basel). 2024 Mar 12;16(6):778. doi: 10.3390/polym16060778 (PMC10974077; doi:10.3390/polym16060778)
Supplement: Supplementary file 1 [file polymers-16-00778-s001.zip › polymers-2874193-supplementary.pdf]

Non-woven fabric thermal-conductive triboelectric nanogenerator via compositing zirconium boride

Xin Wang [1,2], Jinming Liu [2,3], Haiming Chen [2,], Shihao Zhou [2,3] and Dongsheng Mao [2,]**

1 School of Materials Science and Chemical Engineering, Ningbo University, Ningbo 315211, China; wangxin@nimte.ac.cn

2 Key Laboratory of Marine Materials and Related Technologies, Zhejiang Key Laboratory of Marine Materials and Protective Technologies, Ningbo Institute of Materials Technology and Engineering, Chinese Academy of Sciences, Ningbo 315201, China; liujingming@nimte.ac.cn (J.L.); zhoushihao@nimte.ac.cn (S.Z.)

3 Department of Materials Science and Engineering, Zhejiang University of Technology, Hangzhou 310014, China

* Correspondence: chenhaiming@nimte.ac.cn (H.C.); maodongsheng@nimte.ac.cn (D.M.)

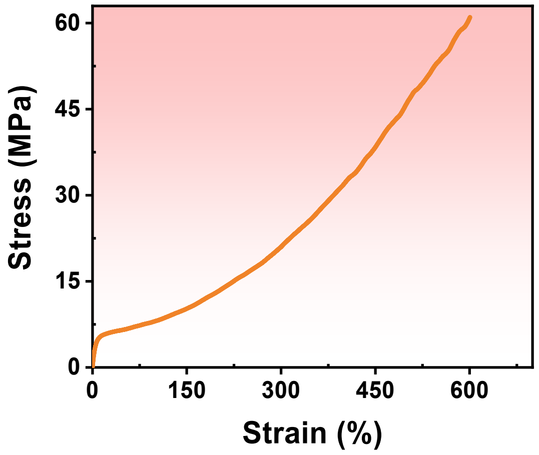

Figure S1. Stress-strain curve of polyurethane. In this study, we successfully constructed an elastomer with high strength and high toughness by combining two incompatible soft segments. The core of this innovative design is that the deformation of the soft phase during the strain process effectively increases the interface energy, thereby significantly improving the mechanical properties of the material. In addition, the forced compatibility phenomenon triggered during the strain process also caused a change in the mixing enthalpy. These two factors increased interfacial energy and changed mixing enthalpy-together contribute significantly to the toughening of elastomers.

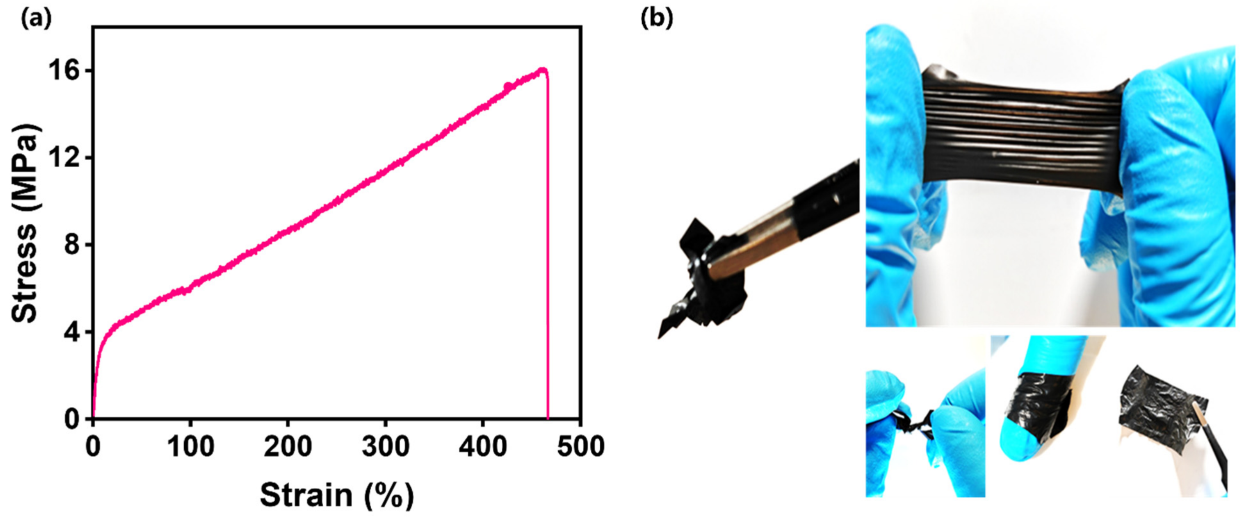

Figure S2. (a) Stress-strain curve of 7.2 vol % ZrB_2/PU non-woven fabric. (b) Digital images of 7.2 vol % ZrB_2/PU non-woven fabric undergo rubbing, stretching and twisting. Although the strength of the spun filaments is lower than that of the bulk one, the strength of the non-woven fabric still reaches 16 MPa, which exceeds that of general polymer non-woven fabrics. And it can be rubbed and stretched randomly without damage, showing excellent practical service capabilities.

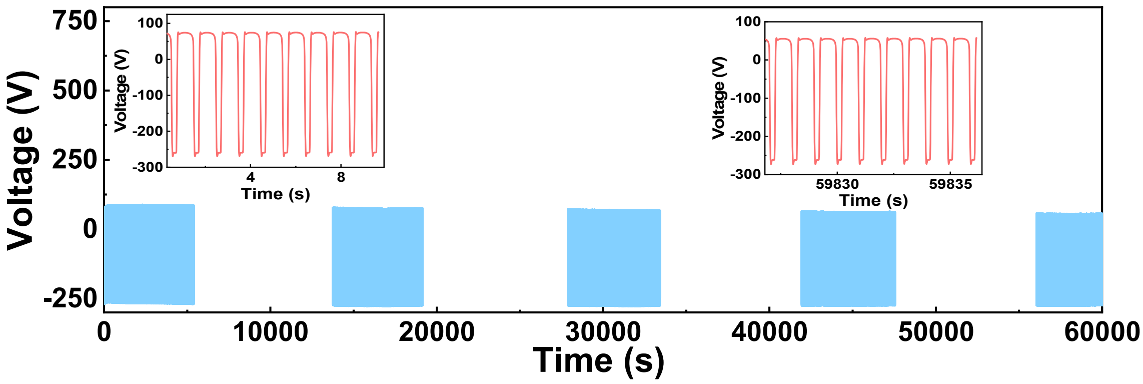

Figure S3. Durability of the TENG by contact seperating the non-woven fabric with ZrB_2 content of 7.2 vol% and PTFE with a frequency of 1 Hz**.** The non-woven fabrics can last more than 60000 cycles without any deterioration in voltage output, indicating that the composite has excellent stability.

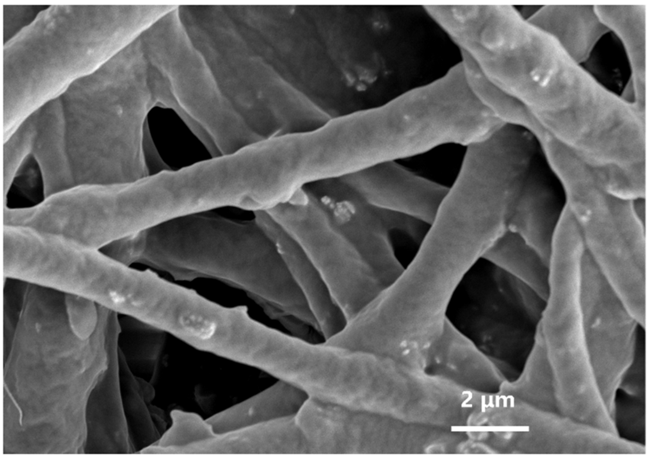

Figure S4. SEM image of non-woven fabric with the 7.2 vol% ZrB_2 which has been undergone 60000 cycles contact-separation. The similar morphology between the one that before and after being contacting shows the fibric structure was not been destroyed, which mainly due to the good elasticity of PU matrix.
